# The Interkingdom Interaction with *Staphylococcus* Influences the Antifungal Susceptibility of the Cutaneous Fungus *Malassezia*

**DOI:** 10.4014/jmb.2210.10039

**Published:** 2022-12-19

**Authors:** Juan Yang, Sungmin Park, Hyun Ju Kim, Sang Jun Lee, Won Hee Jung

**Affiliations:** Department of Systems Biotechnology and Institute of Microbiomics, Chung-Ang University, Anseong 17546, Republic of Korea

**Keywords:** Antifungal drug, co-culture, *Malassezia*, pH, *Staphylococcus*

## Abstract

The skin is a dynamic ecosystem on which diverse microbes reside. The interkingdom interaction between microbial species in the skin microbiota is thought to influence the health and disease of the skin although the roles of the intra- and interkingdom interactions remain to be elucidated. In this context, the interactions between *Malassezia* and *Staphylococcus*, the most dominant microorganisms in the skin microbiota, have gained attention. This study investigated how the interaction between *Malassezia* and *Staphylococcus* affected the antifungal susceptibility of the fungus to the azole antifungal drug ketoconazole. The susceptibility was significantly decreased when *Malassezia* was co-cultured with *Staphylococcus*. We found that acidification of the environment by organic acids produced by *Staphylococcus* influenced the decrease of the ketoconazole susceptibility of *M. restricta* in the co-culturing condition. Furthermore, our data demonstrated that the significant increased ergosterol content and cell membrane and wall thickness of the *M. restricta* cells grown in the acidic environment may be the main cause of the altered azole susceptibility of the fungus. Overall, our study suggests that the interaction between *Malassezia* and *Staphylococcus* influences the antifungal susceptibility of the fungus and that pH has a critical role in the polymicrobial interaction in the skin environment.

## Introduction

Analysis of the microbial community in the human body has revealed that fungi reside with other microbes, such as bacteria, protists, and viruses, within the community. This observation has led to several studies investigating the intra- or inter-kingdom interactions between microbial members influencing skin health and diseases [1-3]. For example, *Pseudomonas aeruginosa* and *Aspergillus fumigatus* often share the host niche, such as the airways of cystic fibrosis patients, and the interkingdom between the microorganisms is likely to occur within the same ecological site. In this scenario, *P. aeruginosa* produces metabolites, such as quorum sensing molecules and siderophores, that possess antifungal activity [4]. Furthermore, studies have reported the effect of bacteria on the antifungal susceptibility of pathogenic fungi. The interaction between *Staphylococcus epidermidis* and *Candida albicans*, co-colonizers of the skin, has also been investigated, especially in a mixed biofilm format. Fluconazole diffusion was retained, but no difference in susceptibility of *C. albicans* to the drug was observed in polymicrobial biofilms [5]. A direct influence of secreted enzymes, such as proteinase, from the major skin-associated fungus *Malassezia* on the biofilm formation of *Staphylococcus aureus*, a noteworthy skin opportunistic bacterial pathogen, was also demonstrated experimentally [6].

*Malassezia* is a commensal fungus found on the skin surfaces of warm-blooded animals, including humans, and comprised of 18 species [7-9]. Several studies based on amplicon sequencing and metagenome analysis demonstrated that *Malassezia* is the most dominant genus in the human skin. This fungus has been associated with various skin diseases, such as seborrheic dermatitis, dandruff, and atopic dermatitis [10, 11]. However, the roles of *Malassezia* in the skin must still be elucidated. Ketoconazole is an imidazole derivative and the most commonly used azole antifungal drug to treat *Malassezia*-associated skin diseases [12-14]. The mechanism of ketoconazole’s action against pathogenic fungi includes inhibiting 14α-lanosterol demethylase, which is encoded by *ERG11*, and converting lanosterol to ergosterol in the ergosterol biosynthesis pathway for fungal membrane synthesis; this results in the accumulation of toxic sterols causing increased membrane stress and growth arrest [15, 16]. Although the effectiveness of ketoconazole is well established, studies have reported that several fungal strains, including *Malassezia*, that have been isolated from clinical specimens were resistant to the drug [17-19]. In general, increased drug efflux, modification of the ergosterol biosynthetic pathway, and drug-targeting enzymes, such as mutation or overexpression of *ERG11*, are the primary cause of azole resistance [20]. Moreover, mutations in other genes in the ergosterol biosynthetic pathway, such as the *ERG2*, *ERG3*, *ERG5*, and *ERG24* genes found in various fungal pathogens, have been associated with azole drug resistance [21, 22].

In this study, we investigated how the presence of *Staphylococcus* influenced the ketoconazole susceptibility of *Malassezia* fungi, particularly *M. restricta*. The interkingdom interaction between *M. restricta* and *S. aureus* or *S. epidermidis* was focused, and the antifungal susceptibility of the fungus against ketoconazole in the presence or absence of bacterial cells was determined. Altered ketoconazole susceptibility of *M. restricta* was observed when the fungal cells were cultured in the presence of *S. aureus* and *S. epidermidis*. To understand the mechanism behind this interkingdom interaction, we biochemically and genetically analyzed how *Staphylococcus* affects the antifungal susceptibility of *M. restricta*.

## Materials and Methods

### Strains and Growth Conditions

*M. restricta* KCTC 27527 [23], *S. aureus* NCTC 8325-4, and *S. epidermidis* KCTC 13172 were obtained from the Korean Collection for Type Cultures (KCTC, Korea) for this study. *M. restricta* KCTC 27527 was maintained in Leeming and Notman agar (LNA; 0.8% bile salt, 0.5% glucose, 0.1% glycerol, 1% peptone, 0.01% yeast extract, 0.05% glycerol monostearate, 1.2% agar, 0.05% Tween 60, and 0.5% whole fat cow milk) medium or modified Dixon medium (mDixon; 3.6% malt extract, 0.6% peptone, 2% bile salt, 1% Tween 40, 0.2% glycerol, and 0.2%oleic acid) [24, 25]. The mDixon media was prepared and adjusted to pH 5.0 or 6.5 with 5.8 M HCl. The *M. restricta* cells were cultured at 34°C for 3 to 4 days. The *Staphylococcus* cells were grown in tryptic soy broth (TSB; 1.7% casein peptone, 0.3% soybean peptone, 0.25% glucose, 0.5% sodium chloride, 0.25% dipotassium phosphate, and 1.5% agar) at 37°C [26].

### Antifungal Susceptibility Test

Antifungal susceptibility was determined by disk diffusion assay on solid media using the top agar plates prepared as described in a previous study with modification [27]. Briefly, mDixon with 0.5% agar containing 2×10^8^ cells/ml of *M. restricta* and 1×10^4^ CFU/ml of *Staphylococcus* cells were poured onto the solid mDixon medium (1.5% agar). After the medium was solidified, 8 mm paper disks containing different ketoconazole concentrations, as described subsequently, were placed on the top surface of the agar medium. The plates were incubated at 34°C for 3 days, and the diameter of the growth inhibition zone was measured to determine *M. restricta*’s susceptibility to ketoconazole susceptibility.

### Analysis of Organic Acids

The concentrations of organic acids in the co-culture medium were determined by high-performance liquid chromatography (Agilent 1100 Series HPLC system; Agilent Technologies, Inc., USA) with a refractive index (RI) detector (Waters 410 RI monitor, Waters Corp., USA) using an Aminex HPX-87H column (300 × 7.8 mm, BioRad Laboratories, Inc., USA) as described in a previous study [28]. After centrifugation of the co-culture broth, the supernatant was passed through a syringe filter (pore size of 0.22 μm). The column was isocratically eluted at 47°C with a flow rate of 0.5 ml min^–1^ using 0.01 N H_2_SO_4_ as the mobile phase.

### Determination of the Ergosterol Contents

Gas chromatography-mass spectrometry (GC-MS) analysis was performed to determine the ergosterol contents in the fungal cells cultured at different pH as described in a previous study [29]. Briefly, *M. restricta* KCTC 27527 cells were cultured in 20 ml mDixon media at 34°C and harvested by centrifugation at 3,500 g for 5 min. Cell pellets were suspended in 600 μl of chloroform-methanol (2:1, v/v). Glass beads (0.4 mm) were added, and the cell suspension was vortexed for 3 min followed by centrifugation at 2,000 g for 15 min. The supernatant was transferred to a new tube and a 0.2 volume of 0.9% NaCl was added, vortexed for 1 min, and centrifuged. The lower phase was transferred to a new tube, then dried and dissolved in chloroform. The GC-MS analysis of the ergosterol content was performed on an Aligent DB-17 column (60 m, 0.32 mm, 0.5 μm film thickness; Agilent Technologies Inc., ) with a GERSTEL multi-purpose sampler MPS fitted with 2L-XT solid phase microextraction (GERSTEL GmbH & Co. KG, Germany), and a 7890A (GC)/5975C (MSD) system (Agilent Technologies Inc.,). A 2 μl sample was injected in split mode at the split ratio of 10:1. The initial temperature of the GC was held at 100°C for 2 min. The temperature was increased to 200°C at the rate of 50°C/min and subsequently raised to 280°C at the rate of 10°C/min, after which it was maintained for 12 min. Ergosterol was detected using a selected ion monitoring mode with an electron ionization energy of 70 eV. The fragment ions at m/z 363 were used for ergosterol quantitation. Concentrations were normalized to the total protein concentration.

### Transmission Electron Microscopy

Transmission electron microscopy (TEM) was performed as described in a previous study [30]. Briefly, *M. restricta* KCTC 27527 cells were cultured in mDixon medium, pH 6.5 or 5.0, at 34°C for 3 days. Cells were fixed with 2.5% glutaraldehyde in 0.1 M sodium phosphate buffer (PBS) (pH 7.4) at 4°C overnight, then washed for 5 min with 0.1 M PBS (pH 7.4) three times at room temperature. Cells were post-fixed with 1.0% osmium tetroxide in 0.1 M PBS (pH 7.4) at 4°C for 1 h, washed with distilled water three times at room temperature for 5 min, and stained with 0.5% uranyl acetate at 4°C overnight. After washing three times for 5 min in distilled water at room temperature, cells were gradually dehydrated with 30%, 50%, 70%, 80%, 90%, and 100% ethanol for 10 min each at room temperature, and finally washed with 100% ethanol twice. The cells were embedded with fresh Spurr’s resin at 4°C overnight, then subsequently for 3 h at room temperature, and polymerized at 70°C overnight. After polymerization, ultrathin (70-nm-thick) sections were prepared. The samples were mounted on a 200-mesh copper grid, stained with 2% uranyl acetate solution, and analyzed using a LIBRA 120 energy-filtering transmission electron microscope (EF-TEM; Carl Zeiss AG, Germany).

### Determination of Intracellular Heme Contents

The fungal cells were cultured in mDixon media at 34°C. Cells were harvested, then washed twice with PBS (pH 7.4), and suspended in lysis buffer containing 10 mM Tris-HCl (pH 8.0) and 150 mM sodium chloride. The fungal cells were lysed using 0.4 mm glass beads and a Beadbeater homogenizer (BioSpec Products, Inc., USA), followed by centrifugation at 13,000 rpm for 3 min at 4°C. The supernatant was used to measure the heme level with a colorimetric analysis using the BioVision hemin assay kit (BioVision Inc., USA) according to the manufacturer’s instructions. The heme concentrations were normalized with the total protein.

## Results

### Malassezia Co-Cultured with *Staphylococcus* Showed Reduced Susceptibility to Ketoconazole

**Fig. 1:**
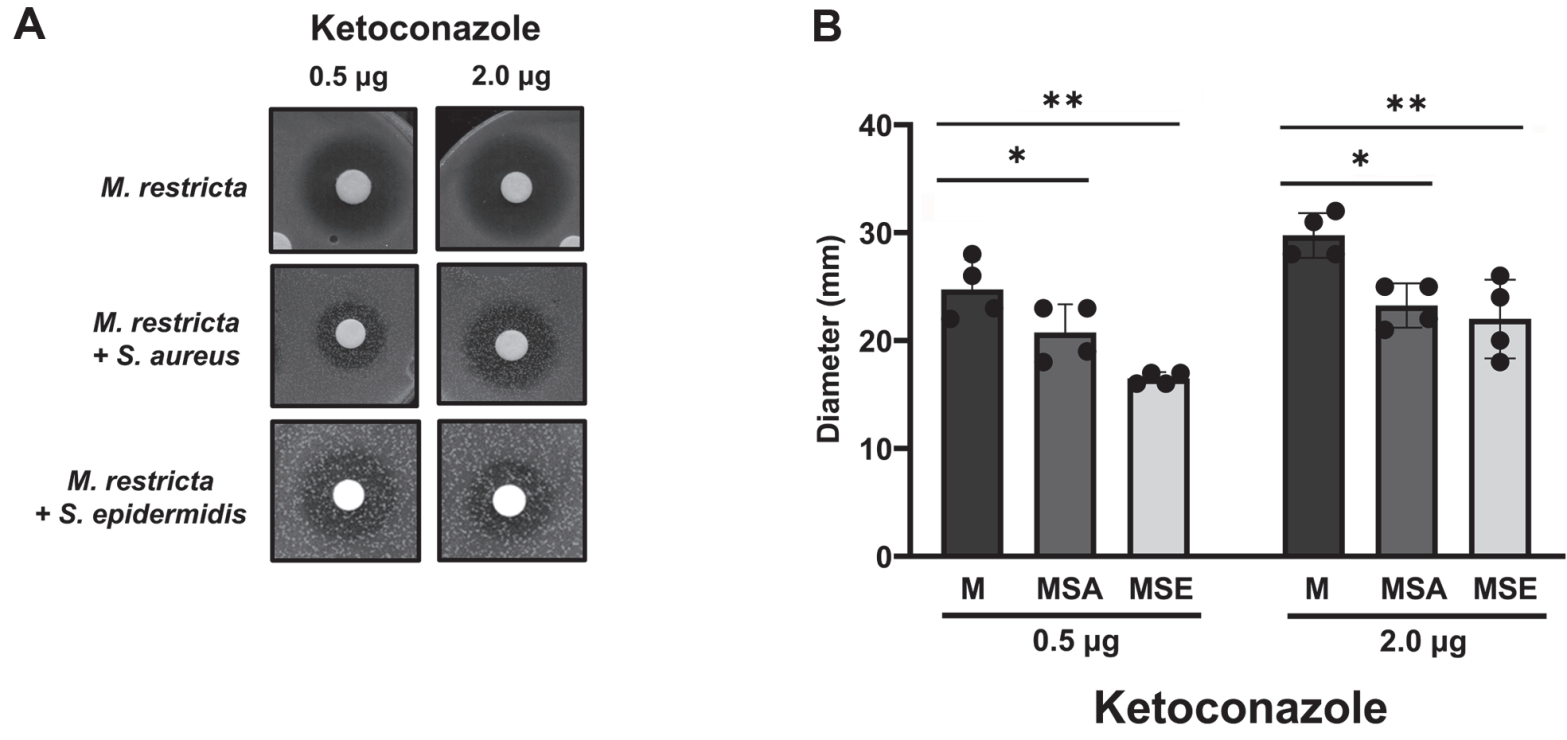
The results of the agar disk diffusion assay. (**A**) Images show different concentrations (0.5 or 2.0 μg per disk) of ketoconazole against *M. restricta* or *M. restricta* co-cultured with *S. aureus* or *S. epidermidis*. (**B**) Diameters of the zone of inhibition on each plate containing different concentrations of ketoconazole were measured and displayed on the y-axis. Four independent experiments were performed, and the values represent averages with standard deviations (*p < 0.05; **p < 0.01).

Among several *Malassezia* species, *M. restricta* was used in our study because the species is the most predominant on human skin [31]. We first tested the growth of *M. restricta* and the *Staphylococcus* species, *S. aureus* and *S. epidermidis*, in either fungal (mDixon) or bacterial (TSB) medium. While *M. restricta* did not grow in the TSB medium, likely due to a lack of sufficient lipid supplement, *Staphylococcus* strains grew well in both media (Fig. S1). Therefore, mDixon medium was selected as a standard medium throughout the study. Next, we tested if the interkingdom interaction between *M. restricta* and *S. aureus* or *S. epidermidis* influenced the susceptibility of the fungus to the azole antifungal drug ketoconazole. Disk diffusion assays were carried out, and the results showed that the zone of inhibition was significantly reduced on the culture plate where *M. restricta* and *S. aureus* or *S. epidermidis* were co-cultured compared to the *M. restricta* axenic culture (Fig. 1). These results indicated that interkingdom interaction between *M. restricta* and *S. aureus* or *S. epidermidis* influenced the susceptibility of the fungal cells to ketoconazole.

### *Staphylococcus* Lowered the pH of the Co-Culture Medium by Producing Organic Acids

**Fig. 2:**
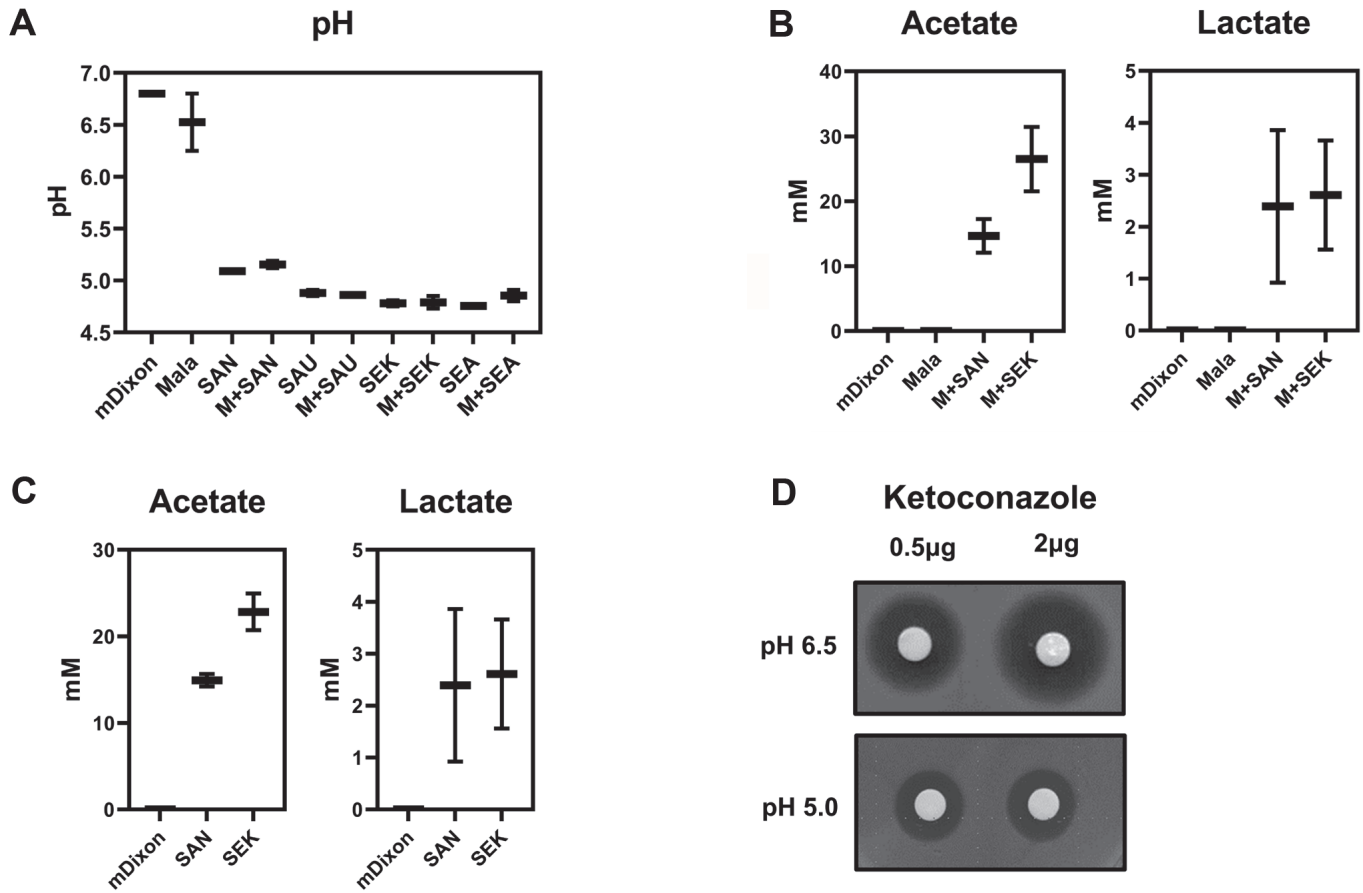
Reduction of pH in the culture medium and production of organic acids by *Staphylococcus*. (**A**) pH of the medium of the *M. restricta* axenic culture or *M. restricta* co-cultured with *S. aureus* or *S. epidermidis* determined after 24 h incubation at 34°C. (**B**) Acetate or lactate production was determined in the medium of the *M. restricta* axenic culture or *M. restricta* co-culture with *S. aureus* NCTC 8325-4 or *S. epidermidis* KCTC 13172 after 24 h incubation at 34°C. (**C**) Acetate or lactate production was determined in the medium of the *S. aureus* NCTC 8325-4 or *S. epidermidis* KCTC 13172 axenic culture after 24 h incubation at 34°C. (**D**) The results of the agar disk diffusion assay. Images show different concentrations (0.5 or 2.0 μg per disk) of ketoconazole against *M. restricta* in different pH mediums. Three independent experiments were performed, and the values represent averages with standard deviations. Mala: *M. restricta* KCTC 27527; SAN: *S. aureus* NCTC 8325-4; SAU: *S. aureus* USA 300; SEK: *S. epidermidis* KCTC 13172; SEA: *S. epidermidis* ACTC 12228.

Next, we investigated the interkingdom interactions between *Malassezia* and *Staphylococcus* that caused the reduced antifungal susceptibility of the fungus. The change in the co-culture medium’s pH was of particular interest; environmental pH has been reported to alter the antifungal susceptibility of pathogenic fungi, such as *C. albicans* [32]. Furthermore, a study suggested that *S. epidermidis* increased the production of organic acids, such as lactic acid, decreasing the pH in mediums containing certain carbohydrates, such as oats [33]. Therefore, the pH changes of the medium’s supernatant were determined when *M. restricta* and *S. aureus* or *S. epidermidis* were co-cultured. While no significant pH change in the *M. restricta* axenic culture medium was observed, a significantly decreased pH (~ 4.7) was observed in the co-culture medium containing *M. restricta* and *S. aureus* or *M. restricta* and *S. epidermidis* (Fig. 2A). Furthermore, pH was decreased in the co-culture medium with different *S. aureus* and *S. epidermidis* strains, suggesting alteration of the pH is a general phenomenon independent of the *Staphylococcus* species and strains (Fig. 2A). However, we noticed that the pH of the co-culture medium was lowered, and the pH of the axenic cultures of either *S. aureus* or *S. epidermidis* were reduced significantly; this indicated that staphylococcal cells altered the pH of the medium no matter if *M. restricta* was present or not (Fig. 2A).

As previously mentioned, we reasoned that the organic acids produced by *Staphylococcus* may be the primary cause of the lowered pH values in the medium. Therefore, we determined the concentration of organic acids, such as acetate, lactate, and succinate, in the co-culture medium after 24 h of incubation. Among the organic acids, the concentrations of acetate and lactate increased markedly in the *M. restricta* and *S. aureus* co-culture medium by 14.6 and 2.4 mM, respectively. In the medium that *M. restricta* and *S. epidermidis* were co-cultured, acetate and lactate were found to be 26.5 and 2.6 mM, respectively (Fig. 2B).

Furthermore, acetate and lactate production levels were determined in the axenic culture of *S. aureus* and *S. epidermidis* to investigate if the increased organic acid concentrations were influenced by the presence of *M. restricta* in the co-culture medium. The concentration of acetate and lactate in the *S. aureus* and *S. epidermidis* axenic culture mediums were similar to that found in the co-culture medium; this suggested that the production of organic acids was solely from the bacterial cells’ metabolism and independent of the presence of *Malassezia* (Fig. 2C).

### Altered Ergosterol Content, Cell Membrane and Wall Thickness, and Intracellular Heme Levels May Contribute to Decreased Ketoconazole Susceptibility of *Malassezia* Cultured in an Acidic pH

Subsequently, we investigated how pH of the medium influenced the susceptibility of *M. restricta* against ketoconazole. *M. restricta* was grown in the medium with a different pH of 5.0 or 6.5, and the cells’ susceptibility to ketoconazole was compared. The results showed that the ketoconazole susceptibility of the *M. restricta* cells grown at pH 5.0 was significantly reduced compared to those grown at pH 6.5. The results confirmed that pH is a critical environmental factor affecting the susceptibility of *M. restricta* against the azole antifungal drug ketoconazole (Fig. 2D).

**Fig. 3:**
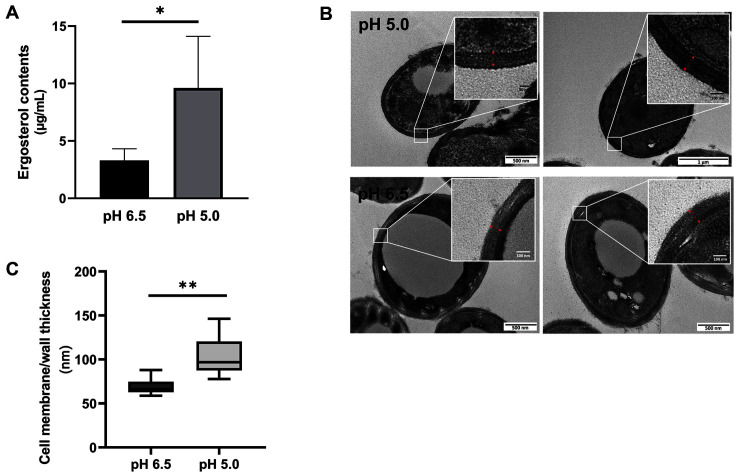
Ergosterol content and the cell membrane and wall thickness were influenced by pH. (**A**) The ergosterol contents in the *M. restricta* cells cultured at different pH mediums were determined by GC-MS. The ergosterol contents were significantly increased in the fungal cells grown at pH 5.0. Four independent experiments were performed, and the values represent averages with standard deviations (*p < 0.05). (**B**) The cell membrane and wall thickness of the *M. restricta* cells grown at different pH mediums were observed by TEM. (**C**) The cell membrane and wall thickness of 50 individual fungal cells grown at pH 5.0 and 6.5. The values represent averages with standard deviations (**p < 0.001).

Several mechanisms are responsible for altering the susceptibility of fungi to azole antifungal drugs; increased ergosterol content is one of them. Indeed, several clinically isolated azole-resistant pathogenic fungi displayed higher ergosterol contents than azole-susceptible strains [34-36]. In this context, we compared the ergosterol contents of *M. restricta* cells grown at pH 5.0 with those grown at pH 6.5. The results showed that the *M. restricta* cells grown at pH 5.0 possessed higher ergosterol content than those grown at pH 6.5; this may contribute to the decreased ketoconazole susceptibility of the *M. restricta* at an acidic pH (Fig. 3A). In addition to measuring the ergosterol contents, we used TEM to observe the *M. restricta* cells’ morphology, cell membrane and wall structure of the cells grown at pH 5.0 and pH 6.5. While no morphological difference was found between *M. restricta* cells grown in the different pH mediums, a significant increase in the cell membrane and wall thickness was observed in the cells grown at pH 5.0 compared with those grown at pH 6.5 (Figs. 3B and 3C).

**Fig. 4:**
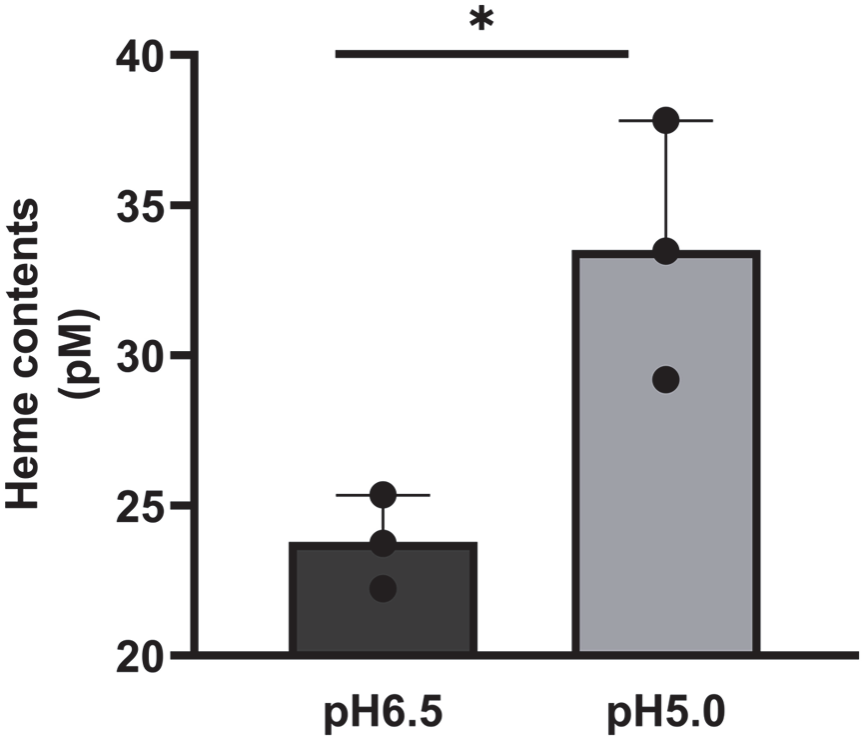
Heme levels in *M. restricta* were influenced by pH. *M. restricta* cells were cultured at pH 5.0 and 6.5 and the intracellular heme levels were determined. Three independent experiments were performed, and the values represented are averages with standard deviations. Statistical significance was calculated using the unpaired two-tailed *t* test. (*p < 0.05).

Ergosterol biosynthesis has been reported to be influenced by iron availability in fungi. Previous studies have shown that iron depletion increased *Aspergillus fumigatus* and *Cryptococcus neoformans*’ fluconazole susceptibility [37-39]. Among the iron-containing metabolites, we were particularly interested in heme because of its involvement in antifungal susceptibility [40-42]. Therefore, we compared the intracellular heme levels in *M. restricta* grown at pH 5.0 with that of cells grown at pH 6.5. The results showed higher heme levels in the *M. restricta* cells cultured in the pH 5.0 medium than that in the pH 6.5 medium; this finding indicated that reduced heme contents might also contribute to the decreased ketoconazole susceptibility of *M. restricta* at an acidic pH (Fig. 4). Overall, our data suggested that *M. restricta* cells’ decreased ketoconazole susceptibility at acidic pH primarily caused by elevated ergosterol content, increased the intracellular heme levels and cell membrane and wall thickness; this implied that environmental pH is critical for *M. restricta*’s antifungal susceptibility and physiology.

## Discussion

This study provided evidence of the interkingdom interaction between *Staphylococcus* and *Malassezia*, predominant bacterial and fungal genera on the human skin, respectively, which influenced the fungus’ susceptibility to the antifungal drug ketoconazole. We demonstrated that the pH reduction mediated by *S. aureus* and *S. epidermidis* decreased *M. restricta*’s ketoconazole susceptibility. The influence of pH on the susceptibility of pathogenic fungi to azole antifungal drugs has been reported previously [43-45]. For example, clinically isolated *C. albicans* strains showed markedly reduced susceptibility to clotrimazole, miconazole, and fluconazole at pH 4.0 than at pH 7.0 [46]. Nevertheless, how pH affects the antifungal susceptibility of the fungi against azole antifungal drugs has not been fully elucidated, and only a few cellular mechanisms have been suggested.

Environmental pH influences the cytoplasmic and cellular organelles’ pH homeostasis and may therefore contribute to changes the susceptibility of a fungal cell to antifungal drugs. In particular, the proper regulation of vacuolar acidification has been reported to be closely linked to azole antifungal treatment in *C. albicans*. Specifically, fluconazole treatment has been demonstrated to disrupt vacuole acidification and trafficking, causing the depletion of ergosterol and deficiency in the vacuole ATPase (V-ATPase) function [47]. V-ATPase is essential in all eukaryotic cells, and *Saccharomyces cerevisiae* mutants lacking *ERG24* exhibited notable growth defects in alkaline conditions while growing relatively well at an acidic pH [47, 48]. The mutant’s growth defects at an alkali pH supported that vacuole acidification is tightly connected with ergosterol synthesis [47]. Furthermore, the *S. cerevisiae* V-ATPase and the *erg24* mutants grew relatively well at pH 4.3, suggesting that the fungal cells are more sensitive to the deficiency in vacuolar acidification and ergosterol synthesis in alkali than acidic growth conditions [47]. Therefore, we speculated that similar mechanisms might exist in *Malassezia*, and ergosterol synthesis and vacuole acidification in the *Malassezia* cells are relatively more vulnerable to azole antifungal drugs at alkali than acidic conditions.

The skin surface’s pH is one of the critical factors for the barrier function of the skin. The optimal pH range of the healthy skin surface typically lies between pH 4.1 and 5.8 (mean 4.9) [49]. Maintaining an acidic pH is contributed by filaggrin degradation pathway, free fatty acids, melanin-containing granules, and sodium-hydrogen exchanger 1 [49]. Maintenance of an acidic pH is also vital for the functions and roles of the stratum corneum, which forms the outermost layer of the skin and is composed of corneocytes filled mainly with keratin proteins and extracellular materials enriched with a lipid matrix [50]. The enzymes involved in synthesizing ceramide, the major lipid component of the stratum corneum, are most active in acidic conditions [51]. An acidic pH also represses the growth and pathogenesis of bacteria, including *Staphylococcus* species, in the skin’s environment [52]. In addition to endogenous mechanisms that contribute to the regulation of pH, microbial products, such as organic and fatty acids from skin microbiota, also contribute acid pH of the skin [53].

In this study, we found that *S. aureus* and *S. epidermis* produced acetate and lactate, reducing the pH of the growth medium; in turn, this contributed to changes in *Malassezia*’s antifungal susceptibility against ketoconazole. Several *in vitro* studies suggested the metabolic characteristics of *S. aureus* in aerobic or anaerobic conditions and have monitored and measured the production of the organic acids to interpret the metabolic remodeling of the bacteria during infections and in different host niches [33, 54]. However, we are not sure if *Staphylococcus* produces organic acids in vivo in relatively large amounts and if this contributes to the acidic pH of the skin. Despite this, the results of this study suggest that the pH of the skin surface affects the outcomes of antifungal treatment.

Our study revealed that intracellular heme levels were increased in *M. restricta* grown in a medium at pH 5.0 compared with a medium at pH 6.5; this may contribute to the decreased ketoconazole susceptibility of the fungus. Intracellular heme content is essential in iron metabolism and homeostasis, and an association between iron metabolism and susceptibility to azole antifungal drugs has been observed in several fungal pathogens. For example, increased fluconazole susceptibilities were found in *C. neoformans* and *A. fumigatus* grown under iron-limited conditions [38, 39]. Moreover, in iron-depleted *C. albicans* cells, downregulation of *ERG11* and reduced ergosterol synthesis were demonstrated [37]. In addition, heme is required for the activity of the damage response protein Dap1, which regulates the stability of the Erg11 protein in *S. cerevisiae* and *C. glabrata* [40, 55]. This observation supports the connection between heme and iron metabolism in ergosterol synthesis and the susceptibility of the fungal cells to the drugs that inhibit the ergosterol synthesis pathway. In this study, we demonstrated that a conserved connection between heme and iron metabolism and azole antifungal drugs exists in *M. restricta*; the fungus increased intracellular heme levels at an acidic pH, which may contribute to increased ergosterol synthesis.

Overall, the results of our study suggested that in interactions between *Staphylococcus* and *M. restricta*, the bacteria reduce the environmental pH by secreting the organic acids acetate and lactate. Reduction of environmental pH altered intracellular heme and ergosterol contents, and increased cell wall and membrane thickness in *M. restricta*, which likely caused decreased ketoconazole susceptibility of the fungus.

## Supplemental Materials

Supplementary data for this paper are available on-line only at http://jmb.or.kr.
