# Supplementary material for: The Interkingdom Interaction with Staphylococcus Influences the Antifungal Susceptibility of the Cutaneous Fungus Malassezia
Source: J Microbiol Biotechnol. 2022 Dec 19;33(2):180–7. doi: 10.4014/jmb.2210.10039 (PMC9998211; doi:10.4014/jmb.2210.10039)
Supplement: Supplementary file 1 [file jmb-33-2-180-supple.pdf]

J. Microbiol. Biotechnol.
https://doi.org/10.4014/jmb.2210.10039

Supplementary Figure

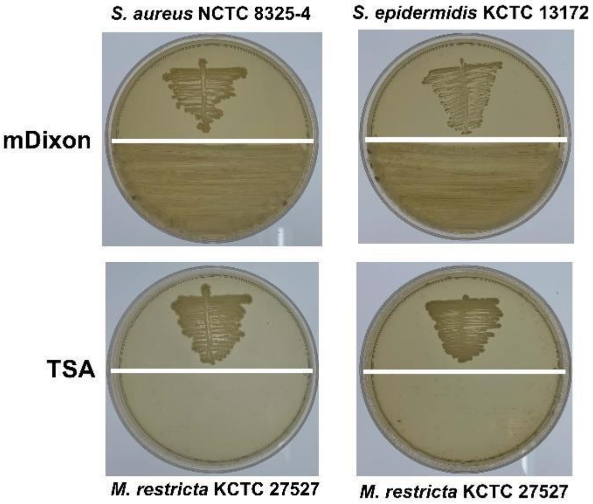

Figure S1. *M. restricta* and *Staphylococcus* species, *S. aureus* and *S. epidermidis*, were grown the fungal (mDixon) or bacterial (TSB) agar medium. The images were taken after three days incubating at 34◦C.
